# Optic Nerve Coloboma in a Child With Compound Heterozygous USH2A Variants

**DOI:** 10.1155/crig/4667935

**Published:** 2025-03-11

**Authors:** Emily S. Levine, Nidhi D. Shah, Erin M. Salcone

**Affiliations:** ^1^Section of Ophthalmology, Department of Surgery, Dartmouth Hitchcock Medical Center, Lebanon, New Hampshire, USA; ^2^Department of Pediatrics, Dartmouth Hitchcock Medical Center, Lebanon, New Hampshire, USA

**Keywords:** coloboma, genetics, retinitis pigmentosa, USH2A, usher syndrome

## Abstract

We present a case of an optic nerve coloboma in a 10-month-old girl found to have compound heterozygous USH2A variants. There were no other dysmorphic features or ocular developmental anomalies. To our knowledge, this is the first report in literature of a concomitant optic nerve coloboma in a case of nonsyndromic retinitis pigmentosa related to USH2A variants.

## 1. Introduction

Patients with Usher syndrome II due to autosomal recessive USH2A variants typically present with moderate-to-severe nonprogressive congenital sensorineural deafness and develop retinitis pigmentosa in the first to second decade of life. There are also variants in the USH2A gene that cause a nonsyndromic retinitis pigmentosa, that is, not associated with sensorineural hearing loss [1]. As retinitis pigmentosa represents a spectrum of retinal degenerative disorders, the entity can coincide with other developmental anomalies of the eye, especially in the context of a syndrome. Reported here is a rare case of optic nerve coloboma in a patient found to have USH2A-related nonsyndromic retinitis pigmentosa upon genetic testing.

## 2. Case Report

A 10-month-old girl was referred to the Dartmouth Hitchcock pediatric ophthalmology clinic for evaluation of a possible eye turn. Examination showed good fix and follow behavior of both eyes but possibly slower response on the left. The eyes were both soft to palpation. Pupils were briskly reactive and symmetric with no relative afferent pupillary defect. Extraocular movements were full in each eye. Alignment was notable for a left esotropia of about 10 prism diopters by the Krimsky test without refixation on cover–uncover. Retinoscopy yielded a cycloplegic refraction of +3.50 sphere bilaterally.

**Figure 1:**
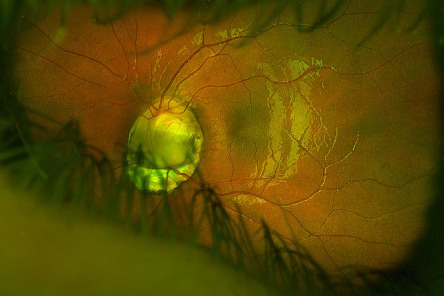
Color fundus photograph of the left retina reveals a large colobomatous left optic nerve.

Hand-held slit lamp exam of the anterior segments of both eyes was normal. Dilated fundus examination revealed a normal appearing right optic nerve with sharp margins. The left eye had a deeply excavated optic nerve head coloboma, with a diameter about 3 times the normal size and with a healthy-appearing neuroretinal rim (Figure 1). There was a small area of mild hypopigmentation just inferior to the right nerve, suggestive of a forme fruste coloboma. Both foveae appeared well developed. No chorioretinal coloboma was seen in bilateral peripheral retinae, and there was no suggestion of a pigmentary retinopathy. Retinal vasculature was normal.

The patient was born full term by spontaneous vaginal delivery following prolonged rupture of membranes after an uncomplicated pregnancy. There were no dysmorphic features. There were possible mild gross motor delays but, otherwise, normal development and growth. Family history was notable for strabismus on both sides, and the maternal great grandmother was noted to have gone both blind and deaf later in life.

The patient underwent genetic testing to evaluate for reportable variants in an 81-gene microphthalmia, anophthalmia, coloboma (MAC) and anterior segment dysgenesis panel, plus analysis for the USH2A gene, with parental segregation (Invitae, San Francisco, CA, USA). In brief, genomic DNA obtained from the submitted samples was enriched for targeted regions using a hybridization-based protocol and sequenced using Illumina technology. Thereafter, reportable variants were analyzed according to published ACMG criteria [2]. The parents had recently undergone prenatal carrier testing as part of a family planning endeavor and were both identified to be carriers of different USH2A gene variants, so USH2A genetic testing was added on after nuanced, extensive conversation with the family about presymptomatic testing for a minor. The MAC panel analysis was nondiagnostic, but the patient was positive for both USH2A variants, a c.4106C > T (p.Ser1369Leu) variant inherited from her father and a c.5438_5443del (p.Ser1813_Ser1815delinsCys) variant inherited from her mother, consistent with a diagnosis of a USH2A-related condition. The patient had passed her newborn hearing screening test, and audiology testing was reported to be within normal limits, suggesting a diagnosis of USH2A-related nonsyndromic retinitis pigmentosa.

## 3. Discussion

To our knowledge, this is the first case of a patient diagnosed with an USH2A-related condition found to have concomitant optic nerve coloboma. There have been few reports describing patients with macular or chorioretinal coloboma and retinal degenerative disorders such as retinitis pigmentosa and retinal dystrophies [3–6], and specifically, one case of Usher syndrome [7]. To date, there are over 2000 reported variants in the USH2A gene [8]. The paternally inherited c.4106C > T variant has been reported in upwards of 20 individuals with USH2A-related conditions while the maternally inherited c.5438_5443del variant has been reported in three individuals with Usher syndrome in previous literature [9–11]. The Invitae genetic testing report predicted one of the variants may disrupt the consensus splice site (c.5438_5443del), whereas the other variant c.4106C > T was not predicted to alter USH2A function, even though it has been seen in trans with known pathogenic variants in affected individuals. USH2A encodes usherin, a transmembrane protein expressed in various tissues including retinal photoreceptors and cochlear hair cells, with a crucial role in photoreceptor survival and cochlear development. In retina photoreceptors, the USH2 complex is required for the maintenance of the periciliary membrane complex that seems to play a role in regulating intracellular protein transport [12].

Despite the rarity of these variants, given that there have never been reported optic nerve colobomas associated with USH2A variants, it is felt this concurrence represents sporadic coincidence rather than genetic association. It should be noted that while the PAX6 gene was adequately covered and no variants of interest were identified, the MAC gene panel may not be an exhaustive list of all coloboma gene targets, as there may be many undiscovered variants in genes that have not yet been associated with coloboma and, therefore, not included in the panel. Although retinitis pigmentosa is expected to develop later in this patient, close monitoring and treatment of strabismus and amblyopia associated with the optic nerve coloboma will be important to maximize visual and general developmental growth. Finally, this case also emphasizes the ethical conversations warranted surrounding presymptomatic genetic testing in a minor.

## Data Availability

Data sharing not applicable to this article as no datasets were generated or analyzed during the current study.

## References

[1] Toualbi L., Toms M., Moosajee M. (2020). USH2A-Retinopathy: From Genetics to Therapeutics. *Experimental Eye Research*.

[2] Richards S., Aziz N., Bale S. (2015). Standards and Guidelines for the Interpretation of Sequence Variants: A Joint Consensus Recommendation of the American College of Medical Genetics and Genomics and the Association for Molecular Pathology. *Genetics in Medicine*.

[3] Ajmal M., Khan M. I., Neveling K. (2014). A Missense Mutation in the Splicing Factor Gene DHX38 Is Associated With Early-Onset Retinitis Pigmentosa With Macular Coloboma. *Journal of Medical Genetics*.

[4] Daggula D. B., Adusumilli H. B., Penmetsa K. C. (2020). Retinitis Pigmentosa With Bilateral Irido-Fundal Coloboma. *Indian Journal of Ophthalmology*.

[5] Panigrahi P. K., Saurabh K., Roy R. (2023). Unilateral Retinitis Pigmentosa and Macular Coloboma With Fellow Eye Pigmented Paravenous Chorioretinal Atrophy. *Clinical and Experimental Optometry*.

[6] Parmeggiani F., Milan E., Costagliola C. (2004). Macular Coloboma in Siblings Affected by Different Phenotypes of Retinitis Pigmentosa. *Eye*.

[7] Ishaq M., Mukhtar A., Khan S. (2015). Macular Coloboma in a Child With Usher Syndrome. *Journal of Ayub Medical College, Abbottabad*.

[8] Lenassi E., Vincent A., Li Z. (2015). A Detailed Clinical and Molecular Survey of Subjects With Nonsyndromic USH2A Retinopathy Reveals an Allelic Hierarchy of Disease-Causing Variants. *European Journal of Human Genetics*.

[9] Cesca F., Bettella E., Polli R. (2020). Frequency of Usher Gene Mutations in Non-Syndromic Hearing Loss: Higher Variability of the Usher Phenotype. *Journal of Human Genetics*.

[10] Colombo L., Maltese P. E., Castori M. (2021). Molecular Epidemiology in 591 Italian Probands With Nonsyndromic Retinitis Pigmentosa and Usher Syndrome. *Investigative Ophthalmology and Visual Science*.

[11] Karali M., Testa F., Di I. V. (2022). Genetic Epidemiology of Inherited Retinal Diseases in a Large Patient Cohort Followed at a Single Center in Italy. *Scientific Reports*.

[12] Van Wijk E., van der Zwaag B., Peters T. (2006). The DFNB31 Gene Product Whirlin Connects to the Usher Protein Network in the Cochlea and Retina by Direct Association With USH2A and VLGR1. *Human Molecular Genetics*.

